# Uncertainty in Model Predictions of *Vibrio vulnificus* Response to Climate Variability and Change: A Chesapeake Bay Case Study

**DOI:** 10.1371/journal.pone.0098256

**Published:** 2014-05-29

**Authors:** Erin A. Urquhart, Benjamin F. Zaitchik, Darryn W. Waugh, Seth D. Guikema, Carlos E. Del Castillo

**Affiliations:** 1 Department of Earth and Planetary Sciences, Johns Hopkins University, Baltimore, Maryland, United States of America; 2 Department of Geography and Environmental Engineering, Johns Hopkins University, Baltimore, Maryland, United States of America; 3 Ocean Ecology Laboratory, NASA Goddard Space Flight Center, Greenbelt, Maryland, United States of America; DOE Pacific Northwest National Laboratory, United States of America

## Abstract

The effect that climate change and variability will have on waterborne bacteria is a topic of increasing concern for coastal ecosystems, including the Chesapeake Bay. Surface water temperature trends in the Bay indicate a warming pattern of roughly 0.3–0.4°C per decade over the past 30 years. It is unclear what impact future warming will have on pathogens currently found in the Bay, including *Vibrio* spp. Using historical environmental data, combined with three different statistical models of *Vibrio vulnificus* probability, we explore the relationship between environmental change and predicted *Vibrio vulnificus* presence in the upper Chesapeake Bay. We find that the predicted response of *V. vulnificus* probability to high temperatures in the Bay differs systematically between models of differing structure. As existing publicly available datasets are inadequate to determine which model structure is most appropriate, the impact of climatic change on the probability of *V. vulnificus* presence in the Chesapeake Bay remains uncertain. This result points to the challenge of characterizing climate sensitivity of ecological systems in which data are sparse and only statistical models of ecological sensitivity exist.

## Introduction

*Vibrio* spp. bacteria are a threat in many coastal aquatic ecosystems around the world [1]–[4]. In the Chesapeake Bay, the number of annual human *Vibrio* cases of infection has nearly doubled in the past decade [5], [6]. Furthermore, *Vibrio* spp. is frequently detected in shellfish harvested for human consumption during the warm summer months [7]. In general, this seasonality correlates with peak incidence of *Vibrio* disease caused by *Vibrio* spp. bacteria in many coastal regions [8]–[10]. The probability of finding various *Vibrio* spp. in the Bay varies spatially and seasonally, and researchers have modeled these probability patterns as a statistical function of surface water temperature and salinity [11]–[16]. These temperature and salinity-based *Vibrio* models have demonstrated skill for available datasets in the Bay and structurally similar statistical models have been applied to predictions of *V. cholerae*, *V. vulnificus*, and *V. parahaemolyticus* in other regions [1], [4], [17], [18]. The environmental range of *V. vulnificus* can vary by region, but in general the bacteria are found in waters with salinity between 5 and 25 (practical salinity units) and temperature above 15°C [12], [19]–[21].

Recent studies show that surface water temperatures in the Chesapeake Bay have warmed by 0.3–0.4°C per decade over the past 30 years [22], [23]. This trend has resulted in an expansion of the warm season period during which water temperatures are high enough to support *V. vulnificus* growth: the onset of spring time temperatures (>15°C) has advanced by nearly three weeks [22]. Salinity patterns are also sensitive to climate change, as changes in springtime flow of the Susquehanna River - the primary freshwater input to the Bay - can influence salinity throughout the Bay over the *V. vulnificus* growth season. The consensus of climate models is that there will be a rise in winter and spring precipitation in the northern portion of the watershed [24], [25] implying an increase in January to May Susquehanna River steam flow. A study by Gibson and Najjar (2000; [26]) showed that an increase in the January-May Susquehanna stream flow could potentially decrease winter and springtime salinity values by 7% in the upper Chesapeake Bay.

Even though there is considerable uncertainty in the magnitude of projected warming and freshening of the Chesapeake Bay [27], it is valuable to understand how a temperature and salinity sensitive pathogen like *V. vulnificus* might respond to observed and projected trends in these environmental parameters. Here we examine three statistical models of *V. vulnificus* probability of presence that demonstrate skill in predicting *V. vulnificus* probability of presence in Chesapeake Bay. All three models use water surface temperature and salinity as the only predictors, but they differ in their structure and/or in the data used for training and evaluation. One model is the generalized linear model (GLM) of Jacobs et al. (2010; [12]) trained on data collected in the Chesapeake Bay in 2007 and 2008, the second model is also a GLM but trained on a 2011 and 2012 data set, while the third model is generalized additive model (GAM) also trained on the 2011–2012 data. The latter two models are from Urquhart et al. (2014; [15]), where surface temperature and salinity were used to model both the probability of presence and concentration *Vibrio* spp. in the upper Chesapeake Bay. Here we focus just on probability models to enable comparison with Jacobs et al. (2010; [12]), who considered only a probability model. Furthermore, we can evaluate the effect that these differences in structure and training data have on modeled estimates of *V. vulnificus* probability under current climate conditions, which is relevant for pathogen risk assessment and early warning, and consider the implications of these differences for projected *V. vulnificus* risk under climate change.

## Methods

**Figure 1:**
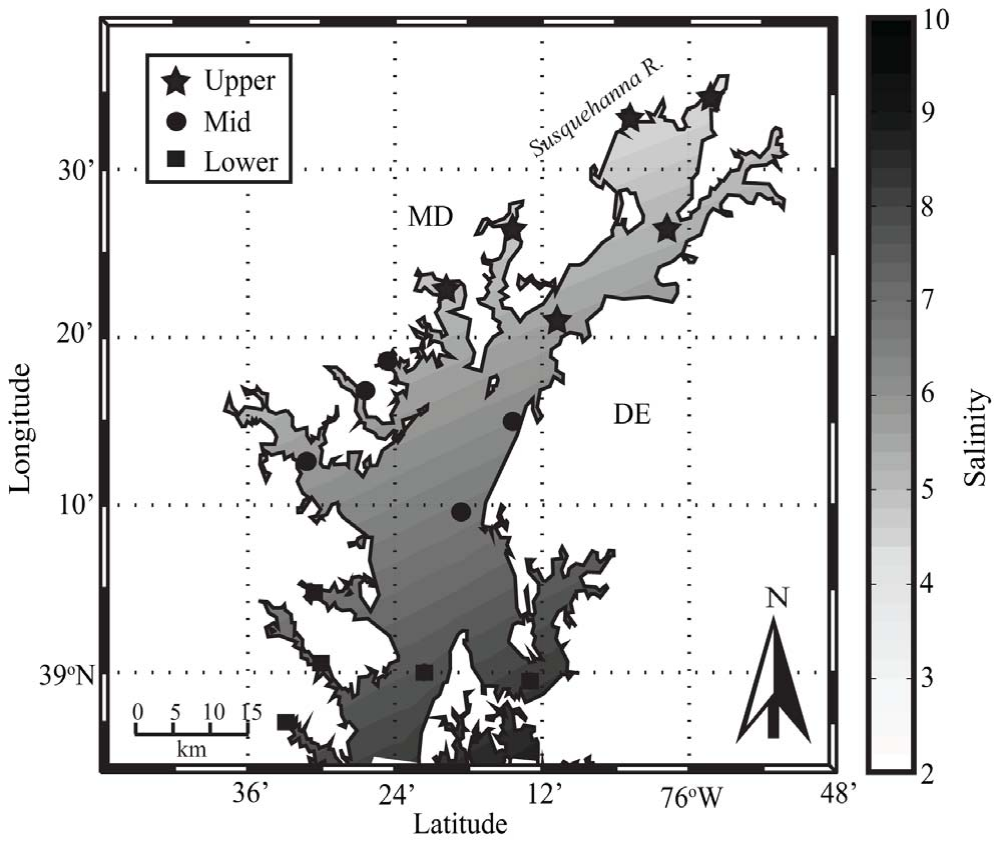
Map of the study area, showing contours of average surface water salinity. Dark markers represent in situ monitoring stations used for each of the subregions in this study: upper (star), mid (circle), and lower (square).

The Chesapeake Bay Estuary, adjacent to the Maryland, Delaware, and Virginia coastline, covers an area of approximately 11,500 km^2^ and is characterized by a sharp north-to-south salinity gradient. Salinity ranges from 0–6 in the northern Bay to 18–30 near the mouth of the Bay. Surface water temperatures follow a seasonal cycle, ranging from local wintertime temperatures of −0.5°C to summertime temperatures of 31°C [28]. The Susquehanna River, the largest and northernmost tributary, accounts for roughly 45% of the yearly freshwater inflow into the Bay. This paper focuses on the upper portion of Chesapeake Bay (Fig. 1). The upper region of the Bay was selected to avoid model predictions outside of the original training data salinity range (salinity >14).

The climatological analysis presented here used historical environmental data collected by the Chesapeake Bay Data Program [29]. Bi-monthly surface water temperature, salinity, and chlorophyll a data were obtained for 16 main stem and tributary monitoring stations (Fig. 1) collected from 1985 through 2013. For salinity, the absolute difference between observed salinity and the *V. vulnificus* optimal salinity value of 11.5 [12] was calculated, and use of deviation from this was used as an explanatory covariate. The 16 monitoring stations were selected based on their geographic location serving as a representation of the upper Chesapeake Bay. In situ data were used to delineate three different salinity zones: upper-upper Bay (hereafter: ”upper region”), middle-upper Bay (hereafter: ”mid region”), and lower-upper Bay (hereafter: ”lower region”). These stations cover the upper main-stem Bay as well as tributary locations, with six stations in the upper region, five stations in the mid region, and five stations in the lower region. Observational data were averaged at monthly intervals for each zone resulting in 337 data records for the upper region and 342 data records for both the mid and lower regions.

These salinity and temperature data were applied to the three statistical *V. vulnificus* probability models available for Chesapeake Bay:

1. NOAA_GLM: The generalized linear model (GLM) of Jacobs et al. (2010; [12]): [*z(V.v)  =  β_0_ + β_1_Temp + β_3_*|*SalnOpt*|, where *β_0_* is the intercept, *β_n_* is the regression coefficient for the independent covariates, *Temp* is daily surface temperature, and |*SalnOpt*| is the absolute distance from optimal salinity of 11.5], which was trained using 235 *V. vulnificus* samples collected during the months of July and October of 2007, and April, July, and October of 2008 and were analyzed by the NOAA Chesapeake Bay Office.
2. JHU_GLM: The GLM of Urquhart et al., (2014; [15]) of the same structural form as the NOAA_GLM [*z(V.v)  =  β_0_ + β_1_Temp + β_3_*|*SalnOpt*|] trained using 148 *V. vulnificus*, surface temperature, and surface salinity samples collected in the upper Chesapeake Bay during the months of July and September of 2011 and March through June of 2012 (Table S1; [15]). Samples were collected by The Johns Hopkins University (JHU) in collaboration with the Maryland Department of the Environment and NASA and were processed at the University of Maryland College Park.
3. JHU_GAM: A generalized additive model (GAM; [30]) trained and evaluated using the same data that were used for JHU_GLM: [*z(V.v)  =  β_0_+s_1_(Temp)+ s_2_(Saln)*, where *s_i_(x_i_)* is a parameter of the smoothing function, and *Saln* is the salinity value].

We included a GAM in addition to the two structurally identical GLMs because GAM models allow a more flexible regression modeling of the transformed response that combine the predictor variables in a nonparametric manner [31]. The NOAA_GLM and JHU_GLM models have the same structure but vary in the training data, whereas the JHU_GLM and JHU_GAM models use the same training data but vary in the structure of model. All models were implemented in logistic form using a “logit” link function for an optimal prediction point and were trained using observational bacteria data transformed to binary presence/absence. Probability of *V. vulnificus* presence was calculated using *p* = *e^z^*/(1+*e^z^*). Diagnostics for each model were performed using Akaike's Information Criterion (AIC) and accuracy (ACC) in an out-of-bag (OOB) cross validation [32]. ACC is defined as ACC  =  (TP+TN)/(P+N) where TP is true positive, TN is true negative, P is the number of presence instances, and N is the number of absence instances.

To explore sensitivity of the *V. vulnificus* models to temperature and salinity, we used a range of surface water temperature (0–40°C) and surface salinity (0–13) values as independent model input. Here the range of model input extends past the range of the in situ temperature (8–31°C) observations, and is constrained to the range of the surface salinity (0–14) observations that were used in the training of the two JHU models. Additionally, historical temperature and salinity data were tested as model input, enabling identification of *V. vulnificus* climatology and seasonal trends. To further assess the geographic distribution of the predicted *V. vulnificus* probability for each method, geospatially-interpolated satellite-derived surface temperature and surface salinity [33], [34] were used to map spatially complete estimates of probability throughout the upper Bay. Interpolated satellite estimates were developed using monthly, level-2 Moderate Resolution Imaging Spectroradiometer (MODIS) surface water temperature (MOD 28) and ocean color (Rrs 412–678) products.

All statistical computations were carried out in the R Statistical Environment version 2.14, using the ‘mgcv’ and ‘stats’ packages, on an Intel Xeon W3580 Processor, 3.33 GHz machine with 12 GB RAM. Computation time for all statistical models was less than one minute.

## Results and Discussion

For model evaluation, goodness of fit and predictive skill for the JHU models were determined using AIC and ACC indices. AIC results indicated that the JHU GAM (145.9) offered better model fit than the JHU GLM (160.4), but performance differences between models were small relative to measurement uncertainty. NOAA GLM model fit using the NOAA training dataset yielded an AIC of 164.3 [12]). A direct comparison of model fit of could not be calculated due to lack of access to NOAA GLM training data. We stress that the difference in training data between the NOAA and JHU models is the primary reason for differences between NOAA_GLM and JHU_GLM, as the models are structurally identical. To predict bacterial presence, selection of an optimal prediction point was required. Rather than setting a prediction point at 0.5 arbitrarily, the prediction point was based on three performance indices: true positive rate, true negative rate, and ACC, yielding an optimal threshold of 0.4 for *V. vulnificus*. To determine the prediction skill of each model, ACC was calculated using the JHU validation dataset (ACC: 0.47, for NOAA GLM, 0.59 for JHU GLM, and 0.60 for JHU GAM). The AIC and ACC values indicated that the JHU models performed significantly better than a null model that only included seasonality as a predictor.

**Figure 2:**
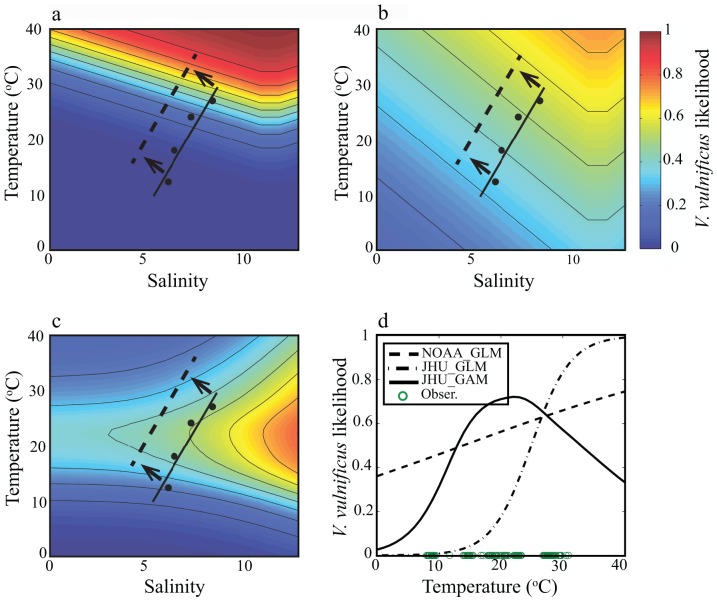
Contour plots of *V. vulnificus* probability with temperature and salinity for (a) NOAA GLM, (b) JHU GLM, and (c) JHU GAM. Black dots represent monthly average (April-July) of in situ conditions; black lines represents in situ trend line, and dashed line represents shift in present day temperature and salinity, (d) Plot of temperature regressed against V.vulnificus probability at 11.5 salinity for each empirical method. Green circles represent the range of temperature observations during bacterium sampling.

Figure 2 shows the relationship between temperature, salinity, and the mean estimate of predicted *V. vulnificus* probability for each of the tested models, with likelihood levels plotted as contour curves. NOAA GLM (Fig. 2a) exhibits a sharp increase in *V. vulnificus* probability with increasing temperatures along the axis of optimal salinity (11.5). Similarly, JHU GLM (Fig. 2b) exhibits a steady increase in probability with higher temperatures, though the rate of change with temperature is less steep than NOAA GLM. In contrast to the GLMs, JHU GAM (Fig. 2c) shows a probability maximum dependent on temperature, indicating a temperature optimum *V. vulnificus* growth above which probability gradually declines. Figure 2d offers an alternative view of predicted *V. vulnificus* probability with temperature, at optimal salinity, including temperature observations during in situ bacteria collection. Furthermore, the wide range of observed temperatures confirms that the declining GAM probability above optimal temperature is a valid model response and not an issue of limited observations at high temperature.

**Figure 3:**
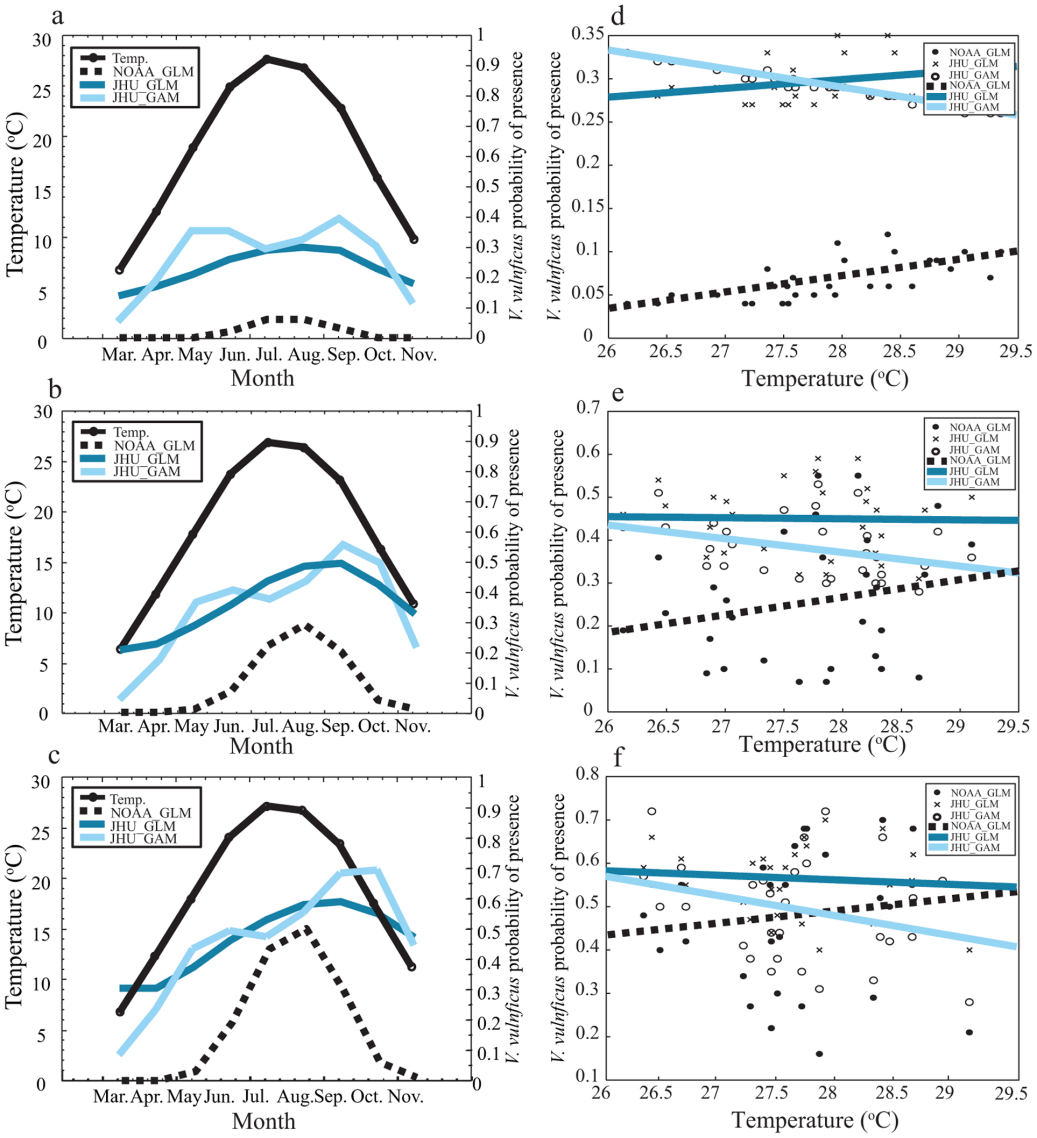
Monthly climatology of temperature and *V. vulnificus* probability for each method in the upper (a), mid (b), and lower (c) regions of the Chesapeake Bay. Peak temperature observations by year versus *V. vulnificus* probability for each method in the upper (d), mid (e), and lower (f) regions of the Chesapeake Bay. Trend lines are included for each method's observations.

These differences in model response also have implications for retrospective or near real-time estimation of risk of *V. vulnificus* presence. Using a 27-year in situ record of temperature and salinity in the upper Chesapeake Bay, we estimated *V. vulnificus* monthly probability of presence according to each statistical model. Fig. 3 shows the climatology of surface water temperature and mean estimate model predictions in each region of the upper Bay for March through November. A southward increase in predicted probabilities for all statistical methods during summer months suggests that distance from optimal salinity plays a role in the spatial distribution of *V. vulnificus* presence. Predicted probabilities are likely lower in the upper region due to decreased salinity and larger deviation from optimal salinity. Seasonal patterns in all regions indicate that NOAA_GLM and JHU_GLM predict highest probabilities during the warmest summertime months. JHU_GAM exhibits a bimodal seasonal pattern with peaks in early and late summer across all regions. These JHU_GAM results are consistent with the temperature dependency shown in Fig. 2c.

The difference in model sensitivity to temperature has implications for characterizing interannual variability in risk. Fig. 3d–f show mean predicted *V. vulnificus* probability for the upper, middle, and lower portions of the study area plotted against annual peak monthly SST for the available historical record. In all three subregions, NOAA_GLM predicts that peak probabilities were highest in warmer years, while JHU_GAM predicts the opposite and JHU_GLM falls in between. We emphasize that these are the mean probability estimates for each model, and that there may not be statistically significant differences between model predictions in any given year. Nevertheless, mean estimates are commonly used to communicate risk and to project trends, so the fact that two comparably high performing models – NOAA_GLM and JHU_GAM – yield opposite mean estimates of the relationship between warm summers and *V. vulnificus* probability is relevant.

The differences in these model response surfaces also have clear implications for projections of *V. vulnificus* probability under climate change. As a simple demonstration, we consider the consensus prediction of warming and freshening of the Bay (dashed line in Figure 2 a–c). NOAA_GLM projects steady or increasing probabilities: freshening moves conditions away from the salinity optimum but this effect is offset by increases in predicted probability with rising water temperatures. The JHU_GLM shows a similar pattern but with lower sensitivity to changing environmental conditions. In contrast, warming only increases predicted probability of *V. vulnificus* presence in JHU_GAM for relatively cool temperatures, representative of spring or fall conditions. Peak summertime temperatures are already above the temperature optimum in this model, so further warming results in a predicted decline in peak summertime *V. vulnificus* probability.

**Figure 4:**
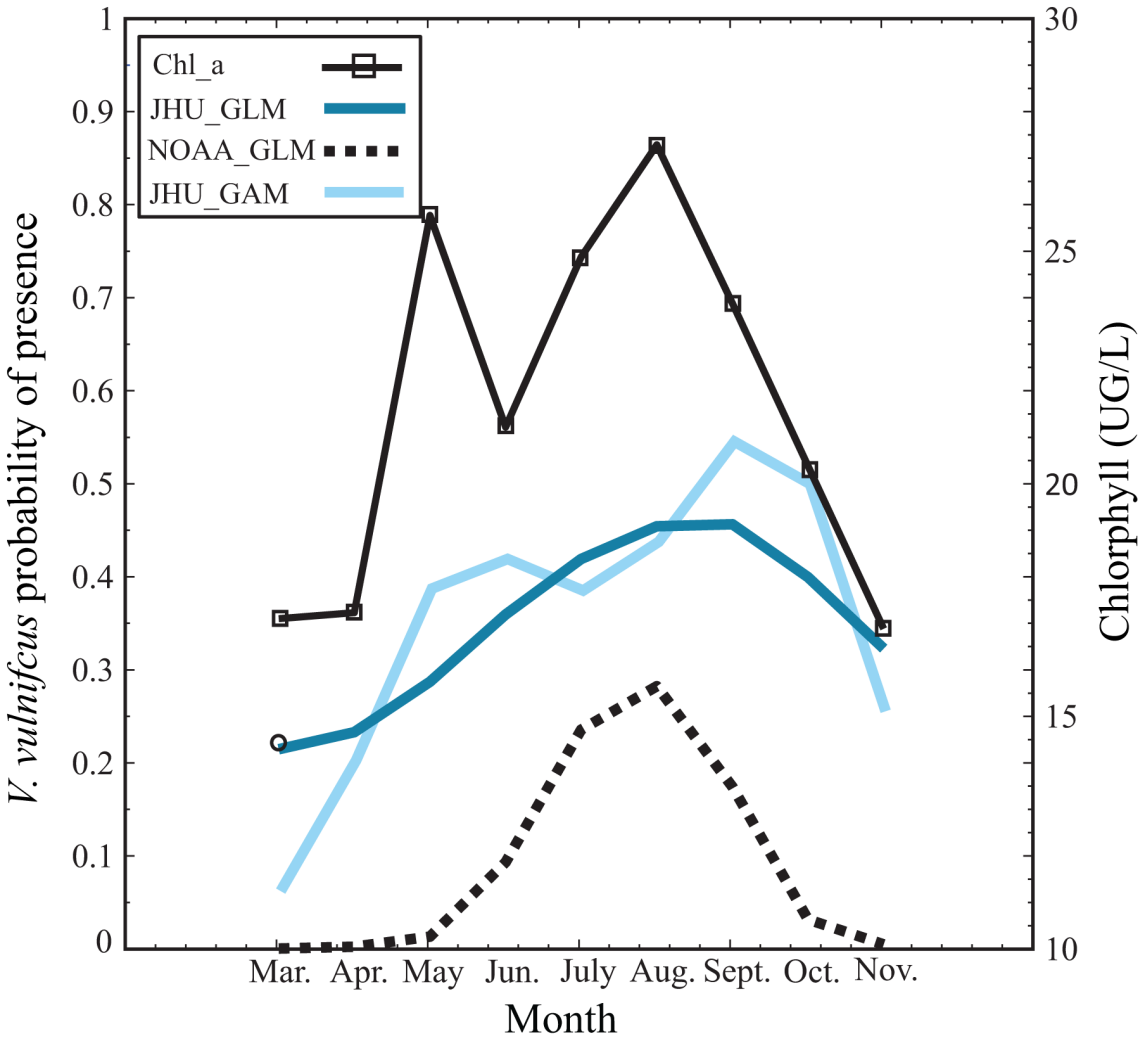
Monthly climatology of Chlorophyll a and *V. vulnificus* probability for each method averaged over the entire upper Chesapeake Bay.

While we cannot presently determine which sensitivity pattern is correct—the JHU_GLM and NOAA_GLM increase with higher temperatures or the JHU_GAM decline under warmest conditions—the JHU_GAM behavior might indicate that present-day summertime water temperatures are already above the optimal temperature for *V. vulnificus* growth in Chesapeake Bay. Alternatively, the result might be understood in the context of previous studies that have shown *Vibrio* dependence on zooplankton due to attachment and/or *Vibrio's* chitinoclastic activity [35], [36]. Unfortunately we do not have adequate co-located measurements of zooplankton and *V. vulnificus* to include zooplankton in a predictive model. However, we do find that the climatology of Chesapeake Bay Program in situ chlorophyll a concentrations, which generally correlate with zooplankton presence, exhibits a bimodal seasonal pattern with a slight lead over the JHU GAM predicted *V. vulnificus* peaks (Fig. 4).

**Figure 5:**
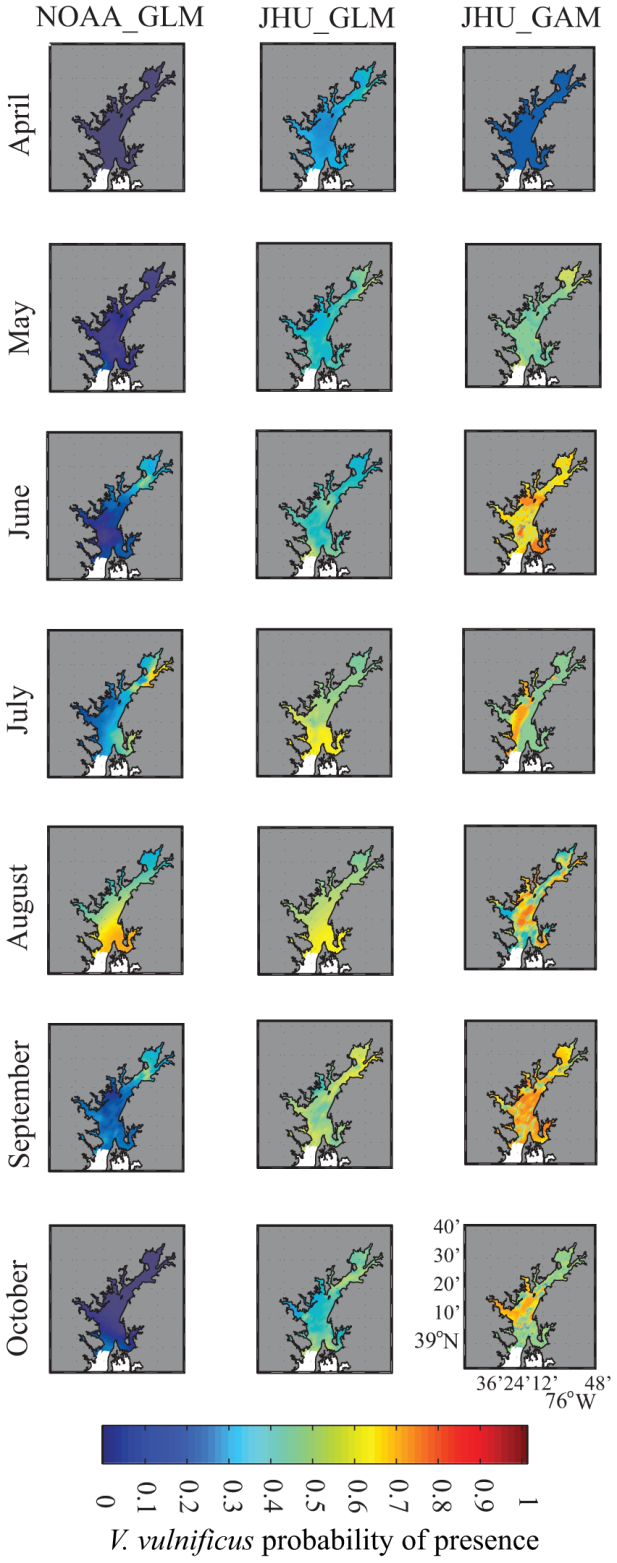
Upper Chesapeake Bay monthly *V. vulnificus* probability hind-casts for April through October 2012, for NOAA GLM, JHU GLM, and JHU GAM methods.

To examine the geographic extent of each methods' predicted *V. vulnificus* probability, monthly interpolated satellite surface water temperature and surface salinity products were used to create spatially complete probability hind-casts for 2012 in the upper Bay (Fig. 5). Consistent with results shown in Fig. 3, these maps show highest predicted probability towards the south of the analysis region, where salinity values are closest to optimum. NOAA_GLM and JHU_GLM both show the most widespread zones of high probability in the warmest summer months, while JHU_GAM predicts higher probabilities at the beginning and end of the warm season. Interesting spatial structures are also apparent in these maps. For example, NOAA_GLM predicts slightly elevated *V. vulnificus* probabilities in the eastern waters of the Chesapeake Bay during warmer months, while JHU_GAM predicts high probability zones in the western Bay during months with lower overall probability (Fig. 5). These patterns likely reflect the Bay's two-layer physical circulation scheme in which we see fresher surface waters along the western shore and saltier waters along the eastern shore of the Bay. The predictions of statistical *V. vulnificus* probability models compared in this study clearly differ in the implied relationships between the structure of this circulation and the location of high *V. vulnificus* risk areas.

## Conclusions

In summary, this study presents a comparison of three statistical ecological habit models for estimating the probability of *V. vulnificus* presence in the upper Chesapeake Bay. We examined individual model sensitivity to climatic variability and change within the upper Bay by assessing model response to a range of temperature and salinity values. We find that the three models differ systematically in the predicted response of *V. vulnificus* probability to high temperatures in the upper Chesapeake Bay.

These results indicate that more data are required to constrain estimates of climate sensitivity of *V. vulnificus* in Chesapeake Bay: statistical models are limited by the paucity of publicly available data from *V. vulnificus* collections and co-located measurements of ecologically relevant variables, and process-based models would require further research on the *V. vulnificus* life cycle in the Bay. Our results also caution against predicting or projecting climate-based changes in *V. vulnificus* exposure risk on the basis of the mean predictions of existing statistical models, as skillful and statistically indistinguishable models differ systematically in predicted *V. vulnificus* sensitivity to rising surface water temperature, even within the range of environmental conditions under which the models were trained.

The challenges facing *V. vulnificus* modeling in Chesapeake Bay are not unique. Indeed, predictive capabilities for climate sensitivity of many pathogens are limited to statistical models based on scarce data. Other recent studies [37]–[38] emphasize that the inadequacy of available data hamper climate change projections for a diversity of waterborne pathogen systems in many regions. In the case of *V. vulnificus* in Chesapeake Bay we have a specific example of closely related modeling efforts that suggest systematically different impacts of climate change due to differences in model structure—i.e., the difference between JHU_GLM and JHU_GAM—and training data—i.e., the difference between JHU_GLM and NOAA_GLM. These kinds of structural comparisons of statistical models, however, are not always performed in studies of climate sensitivity in ecological systems. The results of this study suggest that such model comparisons can be quite important when evaluating uncertainty in climate-based predictions and projections.

## Supporting Information

Table S1 In situ training dataset used for JHU_GLM and JHU_GAM likelihood models.
(TXT)

## References

[1] Baker-Austin C, Trinanes JA, Taylor NG, Hartnell R, Siitonen A, et al (2012) Emerging *Vibrio* risk at high latitudes in response to ocean warming. Nature Climate Change 3: 73–77.

[2] Deepanjali A, Kumar HS, Karunasagar I (2005) Seasonal variation in abundance of total and pathogenic *Vibrio parahaemolyticus* bacteria in oysters along the southwest coast of India. Applied and Environmental Microbiology 71: 3575–3580. 16000764 10.1128/AEM.71.7.3575-3580.2005 PMC1169033

[3] Cantet F, Hervio-Heath D, Caro A, Le Mennec C, Monteil C, et al. (2013) Quantification of *Vibrio parahaemolyticus*, *V. vulnificus* and *V. cholerae* in French Mediterranean coastal lagoons. Research in Microbiology. 10.1016/j.resmic.2013.06.005 PMC4073583 23770313

[4] Oberbeckmann S, Fuchs BM, Meiners M, Wichels A, Wiltshire KH, et al (2012) Seasonal dynamics and modeling of a *Vibrio* community in coastal waters of the North Sea. Microbial Ecology 63: 543–551. 22202887 10.1007/s00248-011-9990-9

[5] Maryland Department of Health (2013) Cases of selected notifiable conditions reported in Maryland. URL http://phpa.dhmh.maryland.gov/SitePages/disease-conditions-count rates.aspx.

[6] Virginia Department of Health (2013) Virginia reportable disease surveillance data. URL http://www.vdh.virginia.gov/Epidemiology/Surveillance/SurveillanceData.

[7] de Magny GC, Murtugudde R, Sapiano MR, Nizam A, Brown CW, et al (2008) Environmental signatures associated with cholera epidemics. Proceedings of the National Academy of Sciences 105: 17676–17681. 10.1073/pnas.0809654105 PMC2584748 19001267

[8] Klontz KC, Lieb S, Schreiber M, Janowski HT, Baldy LM, et al (1988) Syndromes of *Vibrio vulnificus* infections. Clinical and epidemiologic features in Florida cases, 1981–1987. Ann Intern Med 109: 318–23. 3260760 10.7326/0003-4819-109-4-318

[9] Shapiro RL, Altekruse S, Hutwagner L, Bishop R, Hammond R, et al (1998) The role of Gulf Coast oysters harvested in warmer months in *Vibrio vulnificus* infections in the United States, 1988–1996. *Vibrio* Working Group. The Journal of Infectious Diseases 178: 752–759. 9728544 10.1086/515367

[10] Lipp EK, Huq A, Colwell RR (2002) Effects of global climate on infectious disease: the cholera model. Clinical Microbiology Reviews 15: 757–770. 12364378 10.1128/CMR.15.4.757-770.2002 PMC126864

[11] Heidelberg J, Heidelberg K, Colwell R (2002) Seasonality of Chesapeake Bay bacterioplankton species. Applied and Environmental Microbiology 68: 5488–5497. 12406742 10.1128/AEM.68.11.5488-5497.2002 PMC129892

[12] Jacobs J, Rhodes M, Brown C, Hood R, Leigh A, et al (2010) Predicting the distribution of *Vibrio vulnificus* in Chesapeake Bay. NOAA Technical Memorandum NOS NCCOS 112: 1–12.

[13] Wright AC, Hill RT, Johnson JA, Roghman MC, Colwell RR, et al (1996) Distribution of *Vibrio vulnificus* in the Chesapeake Bay. Applied and Environmental Microbiology 62: 717–724. 8593075 10.1128/aem.62.2.717-724.1996 PMC167840

[14] Louis VR, Russek-Cohen E, Choopun N, Rivera IN, Gangle B, et al (2003) Predictability of *Vibrio cholerae* in Chesapeake Bay. Applied and Environmental Microbiology 69: 2773–2785. 12732548 10.1128/AEM.69.5.2773-2785.2003 PMC154498

[15] Urquhart EA, Zaitchik BF, Guikema SD, Haley BJ, Taviani E, et al. (2014) Use of environmental parameters to model pathogenic *Vibrios* in Chesapeake Bay. Journal of Environmental Informatics (Accepted).

[16] de Magny GC, Long W, Brown CW, Hood RR, Huq A, et al (2009) Predicting the distribution of *Vibrio* spp. in the Chesapeake Bay: a *Vibrio cholerae* case study. EcoHealth 6: 378–389. 20145974 10.1007/s10393-009-0273-6 PMC2880626

[17] Eiler A, Johansson M, Bertilsson S (2006) Environmental influences on *Vibrio* populations in north- ern temperate and boreal coastal waters (Baltic and Skagerrak Seas). Applied and Environmental Microbiology 72: 6004–6011. 16957222 10.1128/AEM.00917-06 PMC1563599

[18] Johnson CN, Bowers JC, Griffitt KJ, Molina V, Clostio RW, et al (2012) Ecology of *Vibrio parahaemolyticus* and *Vibrio vulnificus* in the coastal and estuarine waters of Louisiana, Maryland, Mississippi, and Washington (United States). Applied and Environmental Microbiology 78: 7249–7257. 22865080 10.1128/AEM.01296-12 PMC3457101

[19] Colwell R, Kaper J, Joseph S (1977) *Vibrio cholerae, Vibrio parahaemolyticus*, and other *vibrios*: occurrence and distribution in Chesapeake Bay. Science 198: 394–396. 910135

[20] Kaper J, Remmers E, Lockman H, Colwell R (1981) Distribution of *Vibrio parahaemolyticus* in Chesapeake Bay during the summer season. Estuaries 4: 321–327.

[21] Lipp EK, Rodriguez-Palacios C, Rose JB (2001) Occurrence and distribution of the human pathogen *Vibrio vulnificus* in a subtropical Gulf of Mexico estuary. In: The Ecology and Eti- ology of Newly Emerging Marine Diseases,Springer. pp. 165–173.

[22] Austin HM (2002) Decadal oscillations and regime shifts, a characterization of the Chesapeake Bay marine climate. In: American Fisheries Society Symposium. volume 32 , pp. 155–170.

[23] Secor D, Wingate R (2008). A 69-year record of warming in the Chesapeake Bay.

[24] Najjar R, Patterson L, Graham S (2009) Climate simulations of major estuarine watersheds in the Mid-Atlantic region of the US. Climatic Change 95: 139–168.

[25] Hayhoe K, Wake CP, Huntington TG, Luo L, Schwartz MD, et al (2007) Past and future changes in climate and hydrological indicators in the US Northeast. Climate Dynamics 28: 381–407.

[26] Gibson JR, Najjar RG (2000) The response of Chesapeake Bay salinity to climate-induced changes in streamflow. Limnology and Oceanography 45: 1764–1772.

[27] Najjar RG, Pyke CR, Adams MB, Breitburg D, Hershner C, et al (2010) Potential climate-change impacts on the Chesapeake Bay. Estuarine, Coastal and Shelf Science 86: 1–20.

[28] Baird D, Ulanowicz RE (1989) The seasonal dynamics of the Chesapeake Bay ecosystem. Ecological Monographs 59: 329–364.

[29] Chesapeake Bay Program (2013) CBP Water Quality Database (1984-present). URL http://www.chesapeakebay.net/data waterquality.aspx.

[30] Hastie T, Tibshirani R (1986) Generalized additive models. Statistical Science: 297–310. 10.1177/096228029500400302 8548102

[31] Faraway JJ (2004) Extending the linear model with R: generalized linear, mixed effects and non- parametric regression models. CRC press.

[32] Breiman L (1996) Out-of-bag estimation. Technical report, Citeseer.

[33] Urquhart EA, Zaitchik BF, Hoffman MJ, Guikema SD, Geiger EF (2012) Remotely sensed estimates of surface salinity in the Chesapeake Bay: A statistical approach. Remote Sensing of Environment 123: 522–531.

[34] Urquhart EA, Hoffman MJ, Murphy RR, Zaitchik BF (2013) Geospatial interpolation of MODIS- derived salinity and temperature in the Chesapeake Bay. Remote Sensing of Environment 135: 167–177.

[35] Kaneko T, Colwell RR (1973) Ecology of *Vibrio parahaemolyticus* in Chesapeake Bay. Journal of Bacteriology 113: 24–32. 4567138 10.1128/jb.113.1.24-32.1973 PMC251597

[36] Ebi KL (2008) Healthy people 2100: modeling population health impacts of climate change. Climatic Change 88: 5–19. 10.1007/s10584-006-9233-0 PMC7088357 32214561

[37] Hofstra N (2011) Quantifying the impact of climate change on enteric waterborne pathogen concentrations in surface water. Current Opinion in Environmental Sustainability 3: 471–479.

[38] Schets F, Engels G, Evers E (2004) *Cryptosporidium* and *Giardia* in swimming pools in the Netherlands. Journal of Water Health 2: 191–200. 15497815

